# Sound Transmission Loss of Metamaterial Honeycomb Core Sandwich Plate Elastically Connected with Periodic Subwavelength Arrays of Shunted Piezoelectric Patches

**DOI:** 10.3390/ma15113923

**Published:** 2022-05-31

**Authors:** Gongzhuo Yang, Qibai Huang, Mingquan Yang, Yizhe Huang

**Affiliations:** 1 State Key Laboratory of Digital Manufacturing Equipment and Technology, Huazhong University of Science and Technology, Wuhan 430074, China; m202070375@hust.edu.cn (G.Y.); qbhuang@hust.edu.cn (Q.H.); 2 School of Mechanical Engineering, Hubei University of Technology, Wuhan 430068, China; mingquan_yang@outlook.com

**Keywords:** acoustic metamaterial, shunted piezoelectric patches, honeycomb core sandwich plates, sound transmission loss, effective medium method

## Abstract

Honeycomb core sandwich plates are widely used as a lightweight, high-strength sound insulation material. However, they do not perform well in specific frequency bands. Acoustic metamaterials can break the law of mass in specific frequency bands and have high sound transmission loss (STL); however, the resonance frequency is difficult to regulate. To solve this problem, this paper first proposes an infinitely large metamaterial honeycomb core sandwich plate, which can generate newly tuned piezoelectric resonance frequencies, and we study its STL. The structure has piezoelectric patches arranged in sub-wavelength arrays with inductance shunting circuits that are elastically connected to both sides of the honeycomb core sandwich plate. The effective dynamic mass density and effective dynamic bending stiffness of the metamaterial plates were obtained using the effective medium (EM) method. A theoretical model for the numerical calculation of oblique STL and diffuse-field STL was established by the structural bending wave method. The finite element simulation method was used to verify that the metamaterial plates can generate three peaks at 1147 Hz, 1481 Hz and 1849 Hz in oblique or diffuse-field STL curves, which reached 57 dB, 86 dB and 63 dB, respectively, and are significantly better than the plate rigidly connected with piezoelectric sheets and the bare plate with the same mass. In order to better understand the characteristics of STL, the explicit functions of the resonance frequencies were derived. Key influencing factors were analyzed, and the regulation law of new piezoelectric resonance frequencies was clarified.

## 1. Introduction

The honeycomb core sandwich plate is a sandwich-type structure consisting of upper, lower and honeycomb core layers. The honeycomb structure as the core layer is widely used in the fields of aerospace, submarine and naval vessels since it is light weight and high strength [1,2,3,4]. However, there is also the problem of poor sound insulation performance in specific frequency bands [5,6,7]. Scholars have conducted extensive research on the mechanical and acoustic properties of the honeycomb core sandwich plate structure [8,9,10,11,12,13,14,15,16,17,18]. Mechanical equivalent methods of honeycomb sandwich plates mainly include sandwich plate theory [8], Hoff et al. stiffness theory [9], Allen theory [10], Reissner theory [11], etc. [12,13,14]. The STL of honeycomb core sandwich plates was studied by scholars with the wave transfer method [15], structural bending wave method [16], statistical energy analysis (SEA) [17], WFE method [18] and experimental methods [17,18].

Extensive research has proven that the STL of the honeycomb core sandwich plate can be equivalent to a homogeneous plate and that it conforms to the law of mass [6,19,20]. The STL of the honeycomb core sandwich plate is not effectively in specific frequency bands. The traditional way to optimize the STL of plates in airplanes or automobiles is to utilize higher density materials or increase the thicknesses of the plates, which are contrary to the concept of being lightweight. As a sub-wavelength periodic structure with less mass sacrifice, acoustic metamaterials can achieve local resonance in specific frequency bands, break through the law of mass and significantly improve the STL. Thus, this paper focuses on the use of metamaterials to optimize the STL of honeycomb core sandwich plates in specific frequency bands.

Since Liu et al. [21] proposed the local resonant acoustic metamaterials, researchers have been increasingly interested in the research of acoustic metamaterials. Metamaterials are generally considered as artificial composites with sub-wavelength microstructures developed from phonon crystals, which can produce negative effective density and modulus [22], thus, providing excellent low-frequency STL. By adding mass blocks to the thin membrane fixed on the elastic frame and giving the thin membrane a certain initial tension, Yang et al. [23,24,25] proposed that the thin membrane metamaterial can optimize low-frequency STL. 

The tuning of the resonance frequency band can be achieved by controlling the mass blocks and tension. Xiao [26] et al. simulated the STL of an infinite metamaterial plate with periodic resonators by the plane wave expansion method and effective medium method. In summary, compared with membrane-type acoustic metamaterials, thin plate-type acoustic metamaterials can break through the law of mass in the specific frequency range and achieve the optimization of STL, which can be more stable. Therefore, thin plate-type acoustic metamaterials have higher practical application prospects and have a wide range of applications. However, there is a problem that the resonance frequency is single and not easy to be regulated.

The piezoelectric shunt dissipator was first proposed by Forward [27]. Subsequently, Hagood and Van [28] proved that the piezoelectric patches attached to the structure can reduce vibration. With the deepening of scholars’ research on piezoelectric patches, Thorp et al. [29,30] proposed the idea of using periodic piezoelectric patches arrays to design acoustic metamaterials. By designing piezoelectric shunt circuits, the control effect can be easily adjusted. Airoldi and Ruzzene [31] designed a one-dimensional metamaterial with periodic shunt piezoelectric patches, from which negative effective stiffness was found. 

With the further development of research, piezoelectric shunt metamaterial plates have been proven to have excellent performance in the field of acoustics, which can reduce structural sound radiation and promote STL. Zhang [32] et al. designed periodically arranged piezoelectric patches on both sides of the plate and conducted theoretical analysis and simulation verification on the STL using the effective medium (EM) method, which proved that the structure will generate a piezoelectric resonance frequency. Subsequently, Zhang [33,34] finished a further study where piezoelectric patches were periodically attached between orthogonal rib sandwich plates to calculate the STL and far-field sound radiation. Although the elastic connection of piezoelectric patches was considered, the mechanism and influence factors of elastic connected piezoelectric patches were not revealed.

In the current research on acoustic metamaterial plates, elastically connected piezoelectric patches are rarely considered. Only Zhang’s studies [33,34] involved elastically connected piezoelectric patches. In this paper, considering the elastically connected piezoelectric patches and the piezoelectric resonance mechanism, the inductance is used as a piezoelectric patches shunt circuit for further research. This paper mainly completed the work of the following parts. First, the stiffness theory by Hoff et al. was utilized to create the theoretical analysis of the honeycomb core sandwich plate. 

With the piezoelectric shunt theory and effective medium (EM) method, a complete theoretical model of the metamaterial honeycomb core sandwich plate elastic connected with shunted piezoelectric patches was constructed. After that, the results of published papers and the results of finite element simulation (COMSOL) verify the correctness and validity of the theoretical model by comparing the STL of the structure shunted and not shunted piezoelectric plates. Finally, the influence of key parameters on the STL of the structure was studied.

## 2. Theoretical Research

**Figure 1:**
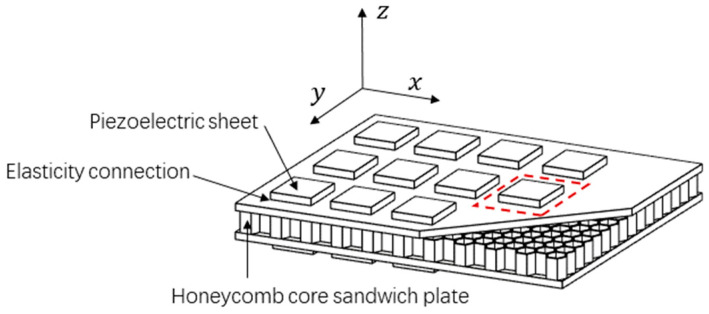
Three-dimensional structure of the metamaterial honeycomb core sandwich plate elastically connected with shunted piezoelectric patches.

The acoustic metamaterial structure designed in this paper is illustrated in Figure 1. The structure elastically connects piezoelectric patches with the inductance shunting circuits to two sides of the honeycomb core sandwich plate in a sub-wavelength cycle. A unit cell consists of two elastically connected piezoelectric patches.

### 2.1. Equivalent Theory of Honeycomb Core Sandwich Plate

The mechanical equivalent theory of honeycomb core sandwich plate includes the sandwich plate theory, Hoff stiffness theory, Allen theory and honeycomb plate theory. For the analysis of large-scale honeycomb core sandwich plate, Hoff stiffness theory, which is effective and precise, can be considered.

**Figure 2:**
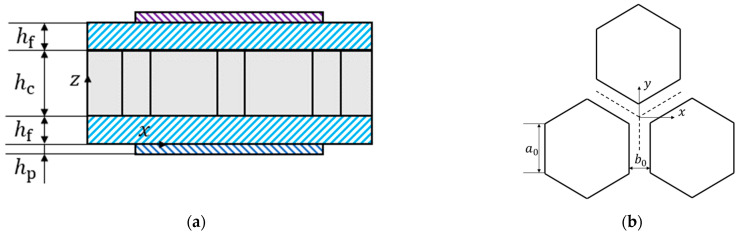
Unit cell structure diagram of metamaterial plate (**a**) a unit cell front view and (**b**) honeycomb core structure.

**Figure 3:**
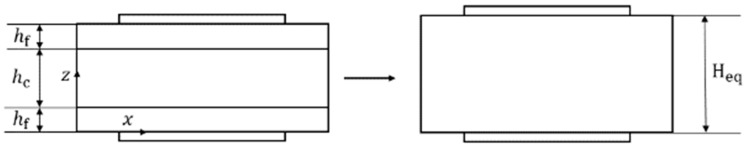
Equivalent schematic of a honeycomb core sandwich plate.

Figure 2a indicates the unit cell structure of metamaterial plate. Figure 2b shows the honeycomb core layer structure. The equivalent stiffness method based on Hoff’s theory is utilized to treat the honeycomb core sandwich plate as an isotropic plate with different thicknesses from the conventional sandwich plate [9].

(1) $$\nu_{\mathrm{eq}}=\nu_{f}$$

(2) $$H_{\mathrm{eq}}=\sqrt{h_{f}^{2}+3{\left(h_{c}+h_{f}\right)}^{2}}$$

(3) $$E_{\mathrm{eq}}=\frac{2E_{f}h_{f}}{H_{\mathrm{eq}}}=\frac{2E_{f}h_{f}}{\sqrt{h_{f}^{2}+3{\left(h_{c}+h_{f}\right)}^{2}}}$$

where $E_{\mathrm{eq}}$, $H_{\mathrm{eq}}$ and $\nu_{\mathrm{eq}}$, respectively, represent the equivalent Young’s modulus, thickness and Poisson’s ratio of the honeycomb core sandwich plate. $E_{f}$, $\rho_{f}$, $\nu_{f}$ and $h_{f}$ are the Young’s modulus, density, Poisson’s ratio and thickness of the surface panel, respectively. $h_{c}$ denotes the thickness of the honeycomb core. $\rho_{c}$ is the core density. According to the law of mass, the equivalent density of the honeycomb core sandwich plate can be expressed as

(4) $$\rho_{\mathrm{eq}}=\frac{k\left(2\rho_{f}h_{f}+\frac{2b_{0}}{\sqrt{3}a_{0}}\rho_{c}h_{c}\right)}{\sqrt{h_{f}^{2}+3{\left(h_{c}+h_{f}\right)}^{2}}}$$

where $\rho_{\mathrm{eq}}$ is the equivalent density of the honeycomb core sandwich plate, and the k=1.5 is taken as the correction factor. Figure 3 can be obtained by equivalenting the unit cell.

### 2.2. Effective Dynamic Mass Density

**Figure 4:**
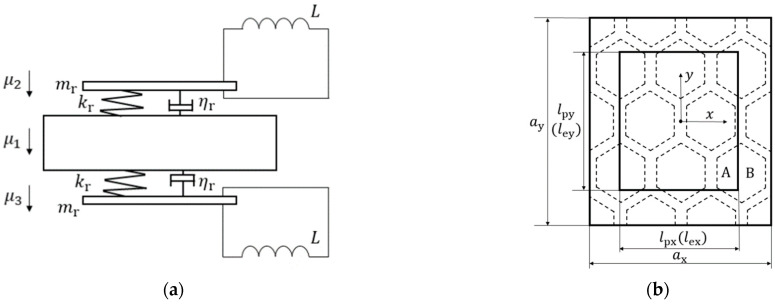
Equivalent schematic diagram of elastically connected piezoelectric sheet (**a**) The front view and (**b**) the top view.

The metamaterial plate designed in this paper is deemed to be distributed in the x–y plane. Since the structure is cyclical, it is sufficient to analyze only one unit cell, which consists of a honeycomb sandwich panel and two piezoelectric sheets elastically connected to the honeycomb sandwich panel. The part containing the elastically connected piezoelectric sheet is region A with the rest being the region B. The length of the unit cell in the *x* direction is $a_{x}$, and the length of the unit cell in the *y* direction is $a_{y}$. Under the sub-wavelength assumption, the elastically connected piezoelectric sheet can be equivalent to a spring–mass resonator. Figure 4 shows the equivalent structure of a unit cell.

Resonator’s concentrated parameters are the equivalent resonator mass $m_{r}$ and the equivalent strength coefficient $k_{r}$.

(5) $$m_{r}=m_{p}$$

(6) $$k_{r}=kA_{p}$$

In (5) and (6), $m_{p}=\rho_{p}l_{\mathrm{px}}l_{\mathrm{py}}h_{p}$ is the mass of the piezoelectric sheet. The density of the piezoelectric sheet is $\rho_{p}$. The length of the piezoelectric sheet in *x* direction is $l_{\mathrm{px}}$. The length of the piezoelectric sheet in *y* direction is $l_{\mathrm{py}}$. The height of the piezoelectric sheet is $h_{p}$. The piezoelectric sheet area is $A_{p}=l_{\mathrm{px}}l_{\mathrm{py}}$. k is the stiffness coefficient per unit area, which equals $2.55\times 10^{4}$. The above Equation (5) can be rewritten as Equation (7).

(7) $$m_{r}=\rho_{p}A_{p}h_{p}$$

The designed resonance frequency $f_{r}$ of the resonator is shown in Equation (8) in which the angular frequency is $\omega_{r}$ [35].

(8) $$f_{r}=\frac{\omega_{r}}{2\pi }=\frac{1}{2\pi }\sqrt{\frac{k_{r}}{m_{r}}}$$

The displacements of the substrate as well as the upper and lower piezoelectric sheets are $\mu_{1}$, $\mu_{2}$ and $\mu_{3}$, respectively. The differential Equations (9) and (10) are established according to Newton’s second law.

(9) $$m_{p}{\ddot{\mu }}_{2}+\eta_{r}\left({\dot{\mu }}_{2}-{\dot{\mu }}_{1}\right)+k_{r}\left(\mu_{2}-\mu_{1}\right)=0$$

(10) $$m_{p}{\ddot{\mu }}_{3}+\eta_{r}\left({\dot{\mu }}_{3}-{\dot{\mu }}_{1}\right)+k_{r}\left(\mu_{3}-\mu_{1}\right)=0$$

The solutions of the differential Equations (9) and (10) are presented in Equations (11) and (12).

(11) $$\frac{\mu_{2}}{\mu_{1}}=\frac{1}{1-\omega^{2}/\left[\omega_{r}^{2}\left(1+i\eta_{r}\right)\right]}$$

(12) $$\frac{\mu_{3}}{\mu_{1}}=\frac{1}{1-\omega^{2}/\left[\omega_{r}^{2}\left(1+i\eta_{r}\right)\right]}$$

According to the effective medium method [26], the equivalent dynamic mass of the spring–mass structure is shown as

(13) $$m_{\mathrm{equiv}}=\frac{\mu_{2}}{\mu_{1}}m_{r}+\frac{\mu_{3}}{\mu_{1}}m_{r}=\frac{2m_{r}}{1-\omega^{2}/\left[\omega_{r}^{2}\left(1+i\eta_{r}\right)\right]}$$

where $\eta_{r}=0.005$, the undamped form is

(14) $$m_{\mathrm{equiv}}=\frac{2m_{r}}{1-\omega^{2}/\omega_{r}^{2}}$$

As per Equations (13) and (14), resonator structure’s equivalent dynamic mass is above zero when $\omega^{2}<\omega_{r}^{2}$. The equivalent dynamic mass is infinitely large when $\omega^{2}=\omega_{r}^{2}$. The equivalent dynamic mass is less than zero when $\omega^{2}>\omega_{r}^{2}$.

In accordance with the effective medium method [26], a honeycomb core sandwich plate can be equivalent to a homogeneous plate with dynamic mass density, and the equivalent dynamic mass of each resonator can be averaged on the substrate to obtain the effective dynamic mass and the effective dynamic mass density.

(15) $$m_{\mathrm{eff}}\left(\omega \right)=\rho_{\mathrm{eq}}SH_{\mathrm{eq}}+m_{\mathrm{equiv}}=\rho_{\mathrm{eq}}SH_{\mathrm{eq}}+\frac{2m_{r}}{1-\omega^{2}/\left[\omega_{r}^{2}\left(1+i\eta_{r}\right)\right]}$$

(16) $$\rho_{\mathrm{eff}}\left(\omega \right)=\left(\rho_{\mathrm{eq}}SH_{\mathrm{eq}}+m_{\mathrm{equiv}}\right)\frac{1}{SH_{\mathrm{eq}}}=\rho_{\mathrm{eq}}+\frac{2\rho_{r}}{1-\omega^{2}/\left[\omega_{r}^{2}\left(1+i\eta_{r}\right)\right]}$$

In Equations (15) and (16), $\rho_{r}=m_{r}/SH_{\mathrm{eq}}$ and $S=a_{x}a_{y}$ is the area of the unit cell. The effective dynamic mass density of the metamaterial plate is a function related to ω, which is different from the static equivalent density.

(17) $$\rho_{\mathrm{st}}=\left(\rho_{\mathrm{eq}}SH_{\mathrm{eq}}+2m_{r}\right)\frac{1}{S}=\rho_{\mathrm{eq}}+2\rho_{r}$$

### 2.3. Effective Dynamic Bending Stiffness

**Figure 5:**
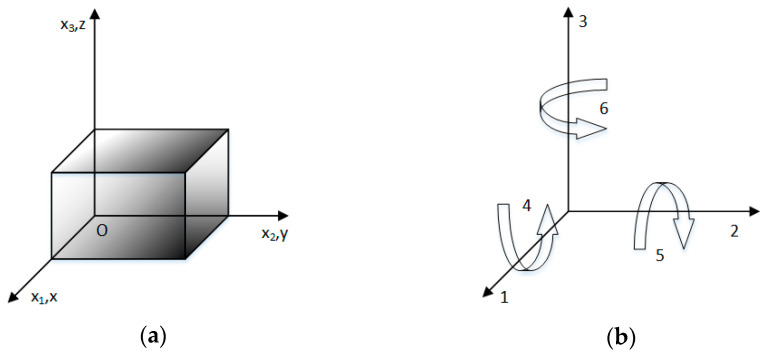
(**a**) The piezoelectric model and (**b**) coordinate system.

First, the piezoelectric patch model and coordinate system are shown in Figure 5.

Since the polarization direction of the piezoelectric patch is along the Z-axis, the constitutive equation of the piezoelectric patch can be described as

(18) $$\left\{S\right\}=\left[s^{E}\right]\left\{T\right\}+{\left[d\right]}^{T}\left\{E\right\}$$

(19) $$\left\{D\right\}=\left[d\right]\left\{T\right\}+\left[\varepsilon^{T}\right]\left\{E\right\}$$

where S and D are the strain tensor and the electric displacement tensor on the piezoelectric plate, respectively, T and E are the stress tensor and the electric field intensity in the piezoelectric plate. $s^{E}$ and $\varepsilon^{T}$ are the flexibility coefficient and dielectric constant of piezoelectric materials, and d is the piezoelectric constant. The specific statements can be obtained from the plane stress state hypothesis

(20) $$\left\{\begin{array}{c} S_{1}\\ S_{2}\\ S_{3}\\ S_{4}\\ S_{5}\\ S_{6} \end{array}\right\}=\left[\begin{array}{c} s_{11}^{E}\\ s_{12}^{E}\\ s_{13}^{E}\\ 0\\ 0\\ 0 \end{array}\begin{array}{c} s_{12}^{E}\\ s_{11}^{E}\\ s_{13}^{E}\\ 0\\ 0\\ 0 \end{array}\begin{array}{c} s_{13}^{E}\\ s_{13}^{E}\\ s_{33}^{E}\\ 0\\ 0\\ 0 \end{array}\begin{array}{c} 0\\ 0\\ 0\\ s_{44}^{E}\\ 0\\ 0 \end{array}\begin{array}{c} 0\\ 0\\ 0\\ 0\\ s_{44}^{E}\\ 0 \end{array}\begin{array}{c} 0\\ 0\\ 0\\ 0\\ 0\\ 2\left(s_{11}^{E}-s_{12}^{E}\right) \end{array}\right]\left\{\begin{array}{c} T_{1}\\ T_{2}\\ T_{3}\\ T_{4}\\ T_{5}\\ T_{6} \end{array}\right\}+{\left[\begin{array}{c} 0\\ 0\\ d_{31} \end{array}\;\begin{array}{c} 0\\ 0\\ d_{31} \end{array}\;\begin{array}{c} 0\\ 0\\ d_{33} \end{array}\;\begin{array}{c} 0\\ d_{15}\\ 0 \end{array}\;\begin{array}{c} d_{15}\\ 0\\ 0 \end{array}\;\begin{array}{c} 0\\ 0\\ 0 \end{array}\right]}^{T}\left\{\begin{array}{c} E_{1}\\ E_{2}\\ E_{3} \end{array}\right\}$$

(21) $$\left\{\begin{array}{c} D_{1}\\ D_{2}\\ D_{3} \end{array}\right\}=\left[\begin{array}{c} 0\\ 0\\ d_{31} \end{array}\;\begin{array}{c} 0\\ 0\\ d_{31} \end{array}\;\begin{array}{c} 0\\ 0\\ d_{33} \end{array}\;\begin{array}{c} 0\\ d_{15}\\ 0 \end{array}\;\begin{array}{c} d_{15}\\ 0\\ 0 \end{array}\;\begin{array}{c} 0\\ 0\\ 0 \end{array}\right]\left\{\begin{array}{c} T_{1}\\ T_{2}\\ T_{3}\\ T_{4}\\ T_{5}\\ T_{6} \end{array}\right\}+\left[\begin{array}{ccc} \varepsilon_{11}^{T} & 0 & 0\\ 0 & \varepsilon_{11}^{T} & 0\\ 0 & 0 & \varepsilon_{33}^{T} \end{array}\right]\left\{\begin{array}{c} E_{1}\\ E_{2}\\ E_{3} \end{array}\right\}$$

Based on the subwavelength precondition, the current generated by the piezoelectric plate is

(22) $$I=\frac{E_{3}h_{p}}{Z}=-A_{p}D_{3}s$$

By solving the equation

(23) $$E_{3}=-\frac{sZA_{p}d_{31}^{2}\left(T_{1}+T_{2}\right)}{h_{p}\left(1+sZC_{p}\right)}$$

Plug into the Equation (20)

(24) $$\left\{\begin{array}{c} S_{1}\\ S_{2}\\ S_{6} \end{array}\right\}=\left[\begin{array}{ccc} s_{11}^{E}-\frac{sZA_{p}d_{31}^{2}}{h_{p}\left(1+sZC_{p}\right)} & s_{12}^{E}-\frac{sZA_{p}d_{31}^{2}}{h_{p}\left(1+sZC_{p}\right)} & 0\\ s_{12}^{E}-\frac{sZA_{p}d_{31}^{2}}{h_{p}\left(1+sZC_{p}\right)} & s_{11}^{E}-\frac{sZA_{p}d_{31}^{2}}{h_{p}\left(1+sZC_{p}\right)} & 0\\ 0 & 0 & 2\left(s_{11}^{E}-s_{12}^{E}\right) \end{array}\right]\left\{\begin{array}{c} T_{1}\\ T_{2}\\ T_{6} \end{array}\right\}$$

Compare to the stress and strain equation of isotropic plates

(25) $$\left\{\begin{array}{c} S_{1}\\ S_{2}\\ S_{6} \end{array}\right\}=\left[-\begin{array}{ccc} \frac{1}{E} & -\frac{v}{E} & 0\\ \frac{v}{E} & \frac{1}{E} & 0\\ 0 & 0 & \frac{1}{G} \end{array}\right]\left\{\begin{array}{c} T_{1}\\ T_{2}\\ T_{6} \end{array}\right\}$$

Piezoelectric sheets with shunting circuits can be equivalent to isotropic plates, and their equivalent Young’s modulus $E_{p}$ and equivalent Poisson’s ratio $v_{p}$ are, respectively, shown

(26) $$E_{p}=\frac{h_{p}\left(1+sZC_{p}\right)}{h_{p}s_{11}^{E}\left(1+sZC_{p}\right)-sZd_{31}^{2}A_{p}}$$

(27) $$v_{p}=-\frac{s_{12}^{E}\left(1+sZC_{p}\right)-sZd_{31}^{2}A_{p}h_{p}^{-1}}{s_{11}^{E}\left(1+sZC_{p}\right)-sZd_{31}^{2}A_{p}h_{p,i}^{-1}}$$

In Equations (26) and (27), s=iω is the Laplace operator ($i=\sqrt{-1}$), where ω=2πf represents the angular frequency. The piezoelectric constant is $d_{31}$. $s_{11}^{E}$ and $s_{12}^{E}$ are the piezoelectric material compliance coefficients at constant electric field intensity. The inherent capacitance of the piezoelectric sheet under constant stress and the impedance of the shunt circuit of the piezoelectric sheet are represented in $C_{p}$ and Z, respectively.

(28) $$C_{p}=\frac{A_{p}\varepsilon_{33}^{T}}{h_{p}}$$

(29) $$Z=R+i\left(\omega L-\frac{1}{\omega C}\right)$$

In (28) and (29), the dielectric constant of the piezoelectric sheet at constant strain is $\varepsilon_{33}^{T}$. The resistance, inductance, and capacitance of shunting circuits are represented by R, L and C, respectively. Since the piezoelectric sheet’s shunting circuit is an inductance, the shunt circuit impedance Z=iωL.

The equivalent dynamic bending stiffness of region A can be obtained by using the classical laminate plate theory [12,32,33].

(30) $$D_{A}=\frac{E_{\mathrm{eq}}H_{\mathrm{eq}}^{3}}{12\left(1-\nu_{\mathrm{eq}}^{2}\right)}+\frac{2E_{p}}{3\left(1-\nu_{p}^{2}\right)}\left({\left(\frac{H_{\mathrm{eq}}}{2}+h_{p}\right)}^{3}-{\left(\frac{H_{\mathrm{eq}}}{2}\right)}^{3}\right)\left(\frac{1}{1-\omega^{2}/\left[\omega_{r}^{2}\left(1+i\eta_{r}\right)\right]}\right)$$

(31) $$D_{B}=\frac{E_{\mathrm{eq}}H_{\mathrm{eq}}^{3}}{12\left(1-\nu_{\mathrm{eq}}^{2}\right)}$$

Thus, a unit cell’s subregion bending stiffness can be described in Equation (32).

(32) $$D\left(x,y\right)=\{\begin{array}{c} D_{A},\left(x,y\right)\in A\\ D_{B},\left(x,y\right)\in B \end{array}$$

According to effective medium method, the unit cell’s effective dynamic bending stiffness is described as

(33) $$D_{\mathrm{eff}}=\frac{D_{A}D_{B}}{\left(1-\alpha \right)D_{A}+\alpha D_{B}}$$

In Equation (33), the area of the piezoelectric sheet as a proportion of the unit cell area is represented by α.

(34) $$\alpha =\frac{l_{\mathrm{px}}l_{\mathrm{py}}}{a_{x}a_{y}}$$

Metamaterial plates are considered to be isotropic homogeneous plates with effective dynamic bending wave numbers.

(35) $$k_{\mathrm{eff}}^{4}=\omega^{2}\frac{\rho_{\mathrm{eff}}H_{\mathrm{eq}}}{D_{\mathrm{eff}}}$$

### 2.4. Sound Transmission Loss of the Metamaterial Plate

**Figure 6:**
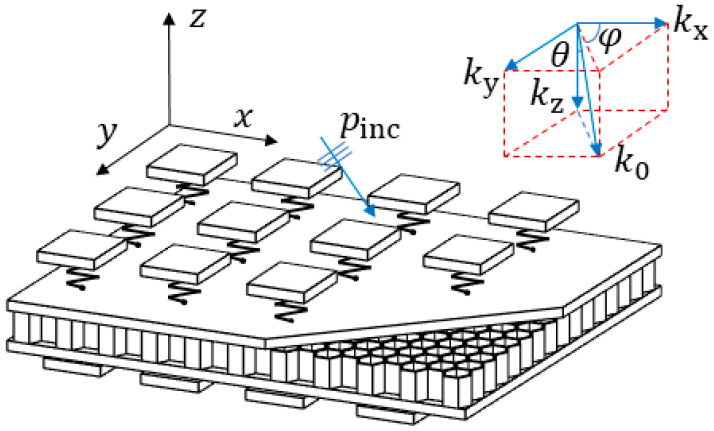
Schematic diagram of the sound transmission.

Figure 6 displays a schematic diagram where a planar acoustic wave goes obliquely into the metamaterial plate. The sinusoidal plane wave hits the metamaterial plate with an elevation angle θ and an azimuth angle φ. The amplitude of the incident wave is $P_{0}$, and the number of the incident waves $k_{0}$ can be decomposed in the *x*, *y* and *z* directions.

(36) $$p_{\mathrm{inc}}\left(x,y,z,t\right)=p_{\mathrm{inc}}\left(x,y,z\right)e^{i\omega t}=P_{0}e^{-i\left(k_{x}x+k_{y}y+k_{z}z\right)}e^{i\omega t}$$

In Equation (36),

(37) $$k_{x}=k_{0}\sin\theta \cos\varphi ,k_{y}=k_{0}\sin\theta \sin\varphi ,k_{z}=k_{0}\cos\theta ,k_{0}=\omega /c_{0}$$

In Equation (37), the $c_{0}$ is the velocity of sound in the air. Due to the distribution of the metamaterial is in the *x*–*y* plane, Equation (38) can be obtained.

(38) $$r=\left(x,y\right),k=\left(k_{x},k_{y}\right)$$

Incident sound pressure that does not change over time can be described as

(39) $$p_{\mathrm{inc}}\left(r,z\right)=P_{0}e^{-irk}e^{-ik_{z}z}$$

Combining with the effective dynamic mass density $\rho_{\mathrm{eff}}$ and the effective dynamic bending stiffness $D_{\mathrm{eff}}$, the vibrational characteristics equation of metamaterial plates excited by incident waves can be described by Equation (40).

(40) $$D_{\mathrm{eff}}\nabla^{4}w\left(r\right)-\rho_{\mathrm{eff}}H_{\mathrm{eq}}\omega^{2}w\left(r\right)=p_{\mathrm{inc}}{\left(r,z\right)}_{z=0}+p_{\mathrm{ref}}{\left(r,z\right)}_{z=0}-p_{\mathrm{tr}}{\left(r,z\right)}_{z=0}$$

where $p_{\mathrm{ref}}$ is the reflected sound pressure, and $p_{\mathrm{tr}}$ is the transmitted sound pressure.

The acoustic characteristics of the infinitely large plate do not depend on the azimuth angel φ [26]. The oblique sound power transmission coefficient can be derived.

(41) $$\tau_{p}\left(\theta ,\varphi \right)=\tau_{p}\left(\theta \right)=\frac{{\left|P_{\mathrm{tr}}\right|}^{2}}{{\left|P_{0}\right|}^{2}}={\left|\frac{2\rho_{0}c_{0}\omega /\cos\theta }{D_{\mathrm{eff}}{\left(k_{0}\sin\theta \right)}^{4}-\rho_{\mathrm{eff}}H_{\mathrm{eq}}\omega^{2}+2i\rho_{0}c_{0}\omega /\cos\theta }\right|}^{2}$$

where $k_{0}\sin\theta$ is the number of trace waves, which is parallel to the plate. The specific derivation process can be referred to from [26,32]. Diffuse-field sound power transmission coefficient [16] is shown in Equation (42).

(42) $$\tau_{\mathrm{diff}}=\frac{\int_{0}^{78^{\circ }}\tau_{p}\left(\theta \right)\sin\theta \cos\theta d\theta }{\int_{0}^{78^{\circ }}\sin\theta \cos\theta d\theta }$$

Sound transmission loss (STL) is obtained as

(43) $$\mathrm{STL}=10\log_{10}\frac{1}{\tau }$$

Equations (41) and (42) are used to obtain the value of τ in Equation (43).

### 2.5. The Finite Element Model

**Figure 7:**
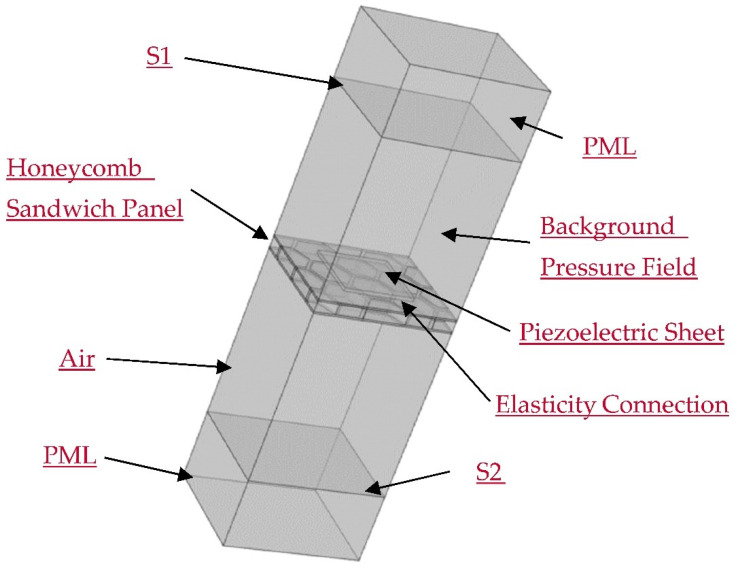
The schematic diagram of the finite element model.

**Figure 8:**
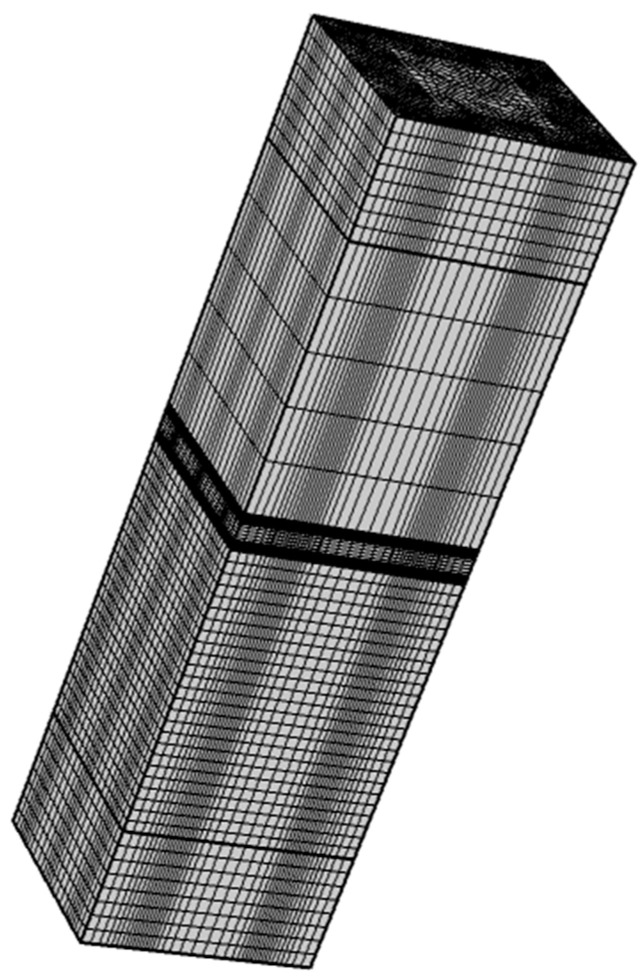
The meshing diagram of the finite element model.

In order to verify the accuracy of the abovementioned theory, this paper establishes a finite element acoustic-structure-piezoelectric coupling model of the designed structure in the finite element software COMSOL. Figure 7 and Figure 8 depict the model diagram and its meshing diagram, respectively. The top and bottom of the model are provided with perfectly matched layers (PML) as the acoustic boundary. Planar incident sound waves are generated by the background pressure field. Piezoelectric sheets are elastically connected on both sides of the honeycomb core sandwich panel. Bloch periodic boundary conditions are set around the model.

The input sound power $W_{\mathrm{in}}$ over the plane $S_{1}$ and the output sound power $W_{\mathrm{out}}$ over the plane $S_{2}$ are calculated to obtain the STL.

(44) $$W_{\mathrm{in}}=\int_{S_{1}}^{\;}\frac{p_{\mathrm{in}}^{2}\cos\theta }{2\rho_{0}c_{0}}ds$$

(45) $$W_{\mathrm{out}}=\int_{S_{1}}^{\;}\frac{p_{\mathrm{out}}^{2}\cos\theta }{2\rho_{0}c_{0}}ds$$

(46) $$\mathrm{STL}=10\log_{10}\frac{W_{\mathrm{out}}}{W_{\mathrm{in}}}$$

## 3. Parameter Settings

The material and geometric parameters of the honeycomb core sandwich panel are shown in Table 1.

The piezoelectric sheet material is set to PZT_5H, and its material characteristic parameters are shown in Table 2. The inductance value L is set to 0.642 H.

## 4. Result Analysis

The length of the bending wave can be obtained via Equations (1)–(4).

(47) $$\lambda_{p}=2\pi {\left(D_{\mathrm{eq}}/\rho_{\mathrm{eq}}H_{\mathrm{eq}}\omega^{2}\right)}^{1/4}=2\pi {\left(\frac{E_{\mathrm{eq}}H_{\mathrm{eq}}^{3}}{12\left(1-\nu_{\mathrm{eq}}^{2}\right)\rho_{\mathrm{eq}}H_{\mathrm{eq}}\omega^{2}}\right)}^{1/4}$$

where $D_{\mathrm{eq}}$ is the equivalent bending stiffness of the honeycomb core sandwich plate.

**Figure 9:**
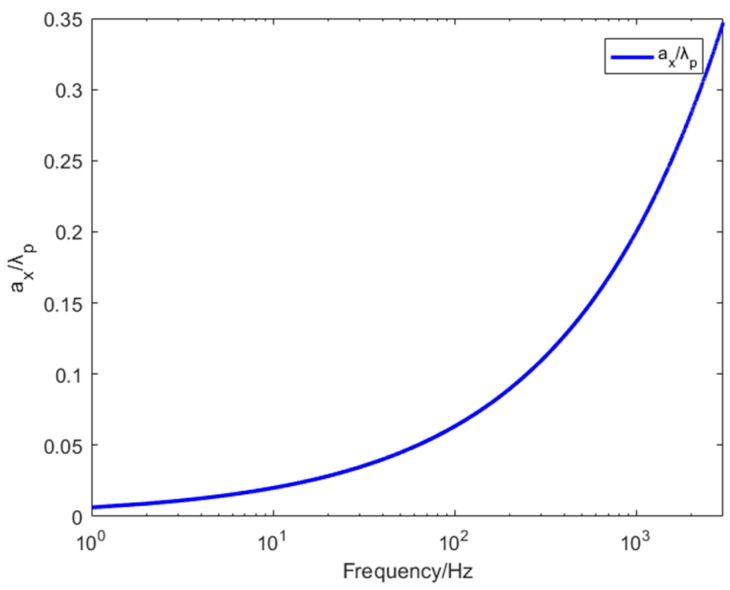
Lattice constant/length of the curved wave.

The results showed in Figure 9, the designed structure meets the sub-wavelength conditions required by the effective medium theory. In other words, the lattice constant $a_{x}$ is much smaller than the length of the bending wave. However, the ratio of two parameters increases with frequency, which also explains that EM theory can be well fitted with finite element simulations in the low-frequency band, and the error will gradually occur in the high-frequency band.

### Effective Dynamic Bending Stiffness and Effective Dynamic Mass Density

**Figure 10:**
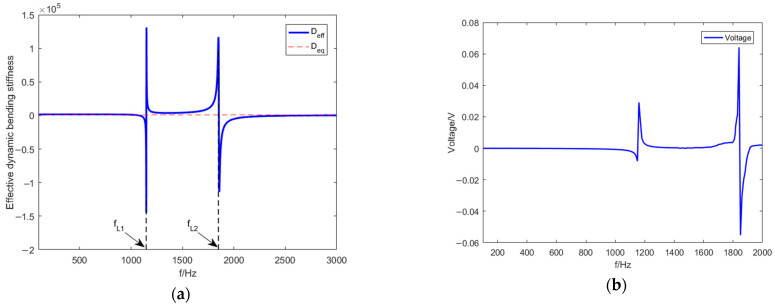
(**a**) Effective dynamic bending stiffness curve. (**b**) Voltage across the inductance.

**Figure 11:**
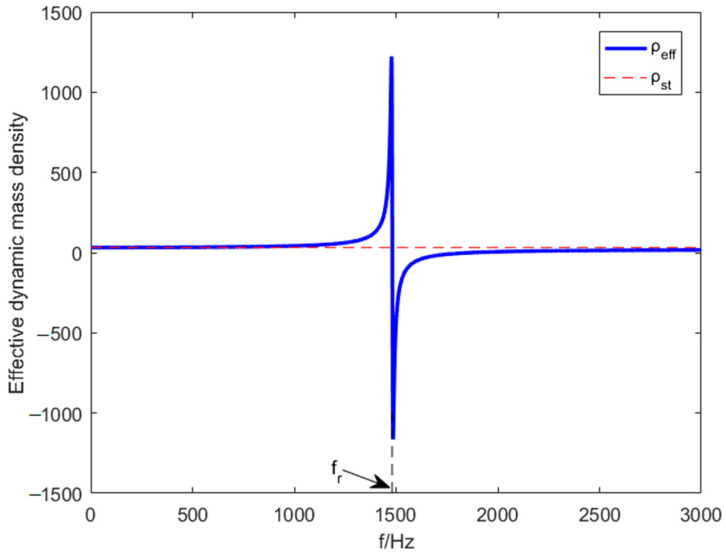
Effective dynamic mass–density curve.

In this section, the periodic metamaterial plate designed herein is proven that to achieve unique dynamic bending stiffness characteristics and dynamic mass density characteristics. Figure 10 and Figure 11, respectively, represent the effective dynamic bending stiffness and the effective dynamic mass density curves changing with frequency when the elevation angle $\theta =30^{\circ }$.

Figure 10a,b show the effective bending stiffness becomes negative when piezoelectric resonance frequency $f_{L1}$ equals 1147 Hz or $f_{L2}$ equals 1849 Hz, which cause the voltage peaks because of the electromechanical coupling. Figure 11 represents the effective mass density becomes negative when mass resonance frequency $f_{r}$ equals 1481 Hz. Negative effective bending stiffness and negative effective mass density are achieved.

**Figure 12:**
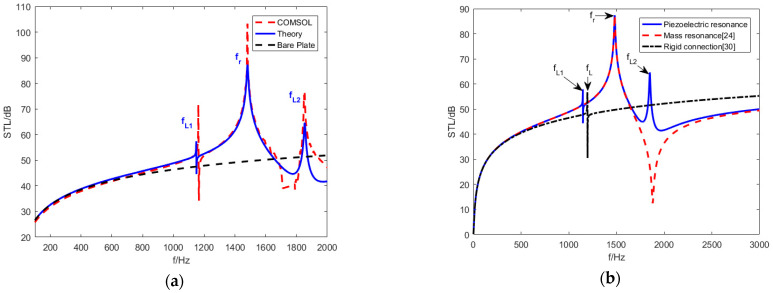
Comparison of theoretical numerical results and FE simulation results. (**a**) Numerical calculations compared to Comsol results. (**b**) STL curve [26,32] under the same conditions.

Figure 12a shows the theoretical numerical results and finite element simulation results. Due to the negative bending stiffness and density, as per the STL curves, three STL peaks are achieved at $f_{L1}$, $f_{L2}$ and $f_{r}$, and the STL near the resonant frequencies is significantly better than the plate with the same weight. Figure 12b reproduces the STL of metamaterial plate rigidly connected piezoelectric sheet studied by Zhang [32] and the STL of mass resonant metamaterial plate studied by Xiao [26] under the same conditions. Positive effective bending stiffness and negative effective mass density were obtained in Xiao’s study. Negative effective bending stiffness and positive effective mass density were obtained in Zhang’s study where piezoelectric resonance frequency $f_{L}$ were proposed [32].

(48) $$f_{L}=\frac{1}{2\pi \sqrt{L\left(C_{p}-\frac{d_{31}^{2}A_{p}/h_{p}}{s_{11}^{E}-\frac{E_{\mathrm{eq}}H_{\mathrm{eq}}^{3}{\left(s_{11}^{E}-s_{12}^{E}\right)}^{2}}{\left(\left(1-\nu_{\mathrm{eq}}^{2}\right)\left(1-\alpha \right)\left({\left(H_{\mathrm{eq}}+2h_{p}\right)}^{3}-{\left(H_{\mathrm{eq}}\right)}^{3}\right)+2E_{\mathrm{eq}}H_{\mathrm{eq}}^{3}\left(s_{11}^{E}-s_{12}^{E}\right)\right)}}\right)}}$$

Substitute the actual values of the variables in Equation (48), the value of $f_{L}$ can be obtained, which equals 1196 Hz. The elastically connected piezoelectric sheet designed in this paper expands piezoelectric resonance frequencies from one to two, which is significantly improved compared with the STL of previous studies. The research in this paper further expands the research of Zhang [33,34] and Zhang [32] to derive the display explicit functions of $f_{L1}$, $f_{r}$ and $f_{L2}$.

The explicit functions of $f_{L1}$ and $f_{L2}$ are derived with the condition that the effective dynamic bending stiffness $D_{\mathrm{eff}}$ approaches 0, which is introduced in Equation (33).

(49) $$f_{L1}=\frac{1}{2\pi }\sqrt{\frac{-X_{2}+\sqrt{X_{2}^{2}-4X_{1}X_{3}}}{2X_{1}}}$$

(50) $$f_{L2}=\frac{1}{2\pi }\sqrt{\frac{-X_{2}-\sqrt{X_{2}^{2}-4X_{1}X_{3}}}{2X_{1}}}$$

where $X_{1}$, $X_{2}$ and $X_{3}$ can be obtained from the Appendix A, and the specific derivation process is shown in the Appendix A.

With the actual values of the variables, the values of $f_{L1}$ and $f_{L2}$ can be obtained, which are 1147 and 1849 Hz, and they confirm the results of a finite element simulation. Depending on Equations (49)–(63), two piezoelectric resonance frequencies are related to the piezoelectric material and substrate parameters.

Under the condition that the mass density $\rho_{\mathrm{eff}}$ approaches 0 is introduced in Equation (16), Equation (51) can be achieved.

(51) $$\rho_{\mathrm{eq}}+\frac{\rho_{r}}{1-\omega^{2}/\left[\omega_{r}^{2}\left(1+i\eta_{r}\right)\right]}\to \infty$$

Equation (52) can be derived from Equation (51).

(52) $$f_{r}=\frac{\omega_{r}}{2\pi }=\frac{1}{2\pi }\sqrt{\frac{k_{r}}{m_{r}}}$$

With the actual values of the variables, the value of $f_{r}$ can be obtained, which equals 1481 Hz, which confirms the results of the finite element simulation.

## 5. The Effects of Crucial Parameters

In this section, in order to explore the impact of elevation angle θ, shunt circuit and area ratio α on structural STL performance, all parameters remain unchanged except the variables selected in each case.

### 5.1. Effect of the Elevation Angle

**Figure 13:**
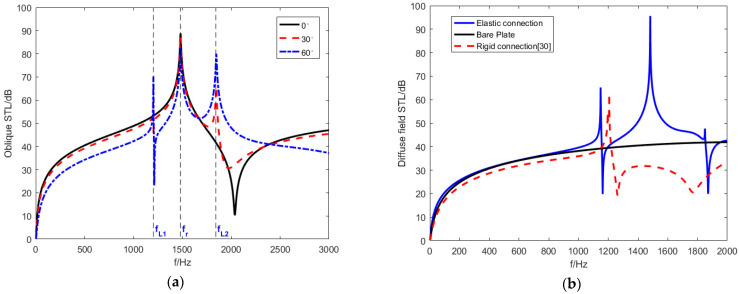
The STL curves at different evelation angles. (**a**) The oblique STL curves (**b**) Diffuse-field STL curves.

Since the object studied is an infinitely large plate, the azimuth angel φ of the planar incident sound wave does not affect the results [26,32]. Figure 13a shows the STL trend with incident waves of different elevation angles. Different elevation angles have a significant impact on the STL performance. 

When the elevation angle θ is $0^{\circ }$, the STL curve only has a mass resonant frequency $f_{r}$, which equals 1481 Hz. According to the analysis of (41), because the elevation angle θ equals $0^{\circ }$ and sinθ equals 0, the effective dynamic bending stiffness $D_{\mathrm{eff}}$ is eliminated. The piezoelectric sheet does not result in piezoelectric resonance and is only affected by the effective mass density $\rho_{\mathrm{eff}}$. Therefore, the STL curve generated by the effective dynamic mass density shows a single-peak characteristic. When the elevation angle θ does not equal $0^{\circ }$. Two new piezoelectric resonance frequencies are generated at $f_{L1}=1147\;\mathrm{Hz}$ and $f_{L2}=1849\;\mathrm{Hz}$. 

Simultaneously, the anti-resonance valley generated by the effective dynamic mass density can be eliminated to some extent. As the elevation angle increases, the resonance frequencies ($f_{r}$, $f_{L1}$ and $f_{L2}$) of the STL remain constant, and the piezoelectric resonance amplitude increases. The piezoelectric resonance frequency bands are widened but the mass resonance frequency band is narrower. The STL in bands other than resonance frequencies is sacrificed. While the elevation angle increases, the coincident frequency decreases [26], which explains why the curve with θ equaling $60^{\circ }$ shows a downward trend after $f_{L2}$, while the other two curves show an upward trend. 

Figure 13b shows the diffuse-field sound power transmission loss and compares to the equal mass bare plates as well as the rigidly connected piezoelectric plates under the same conditions [32]. The results show that the diffuse-field STL of metamaterial plate designed in this paper achieves three resonance frequencies ($f_{L1}$, $f_{L2}$ and $f_{r}$). The diffuse-field STL in the 1000–1800 Hz frequency band near the resonance frequencies is significantly higher than the others; however, the STL amplitude at $f_{L1}$ is increased, and the STL amplitude at $f_{L2}$ is considerably reduced. Under the same conditions, the rigidly connected piezoelectric plate only achieves resonance frequency $f_{L}$ due to lacking the effect of the elastic connection.

### 5.2. Effect of the Shunting Circuit

**Figure 14:**
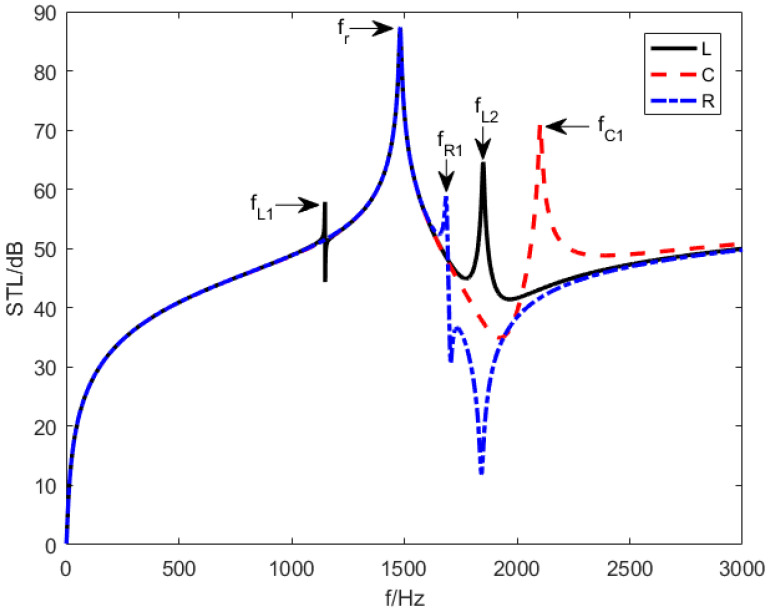
The STL curves under different shunting circuits.

Figure 14 represents the STL of the designed structure with three different shunting circuits including an inductance is involved (Z=iωL), a resistive is involved (Z=R), and a capacitive is involved (Z=i/ωC). R equals 0.642 Ω and C is equivalent to $2\times 10^{-8}F$. As per Figure 14, there are two resonance frequencies in the STL curves when Z=R or Z=i/ωC, including a mass resonance frequency $f_{r}$ at 1481 Hz and a piezoelectric resonant frequency $f_{R1}$ or $f_{C1}$, which are produced by the mass resonance effect and piezoelectric resonance effect, respectively. Explicit functions of $f_{R1}$ and $f_{C1}$ can be derived from Equation (33). However, when an inductance is involved in shunting circuit, the metamaterial plate creates one mass resonance frequency at $f_{r}=1481\;\mathrm{Hz}$ and two piezoelectric resonant frequencies at $f_{L1}=1147\;\mathrm{Hz}$ and $f_{L2}=1849\;\mathrm{Hz}$.

#### 5.2.1. The Inductance Shunting Circuit

**Figure 15:**
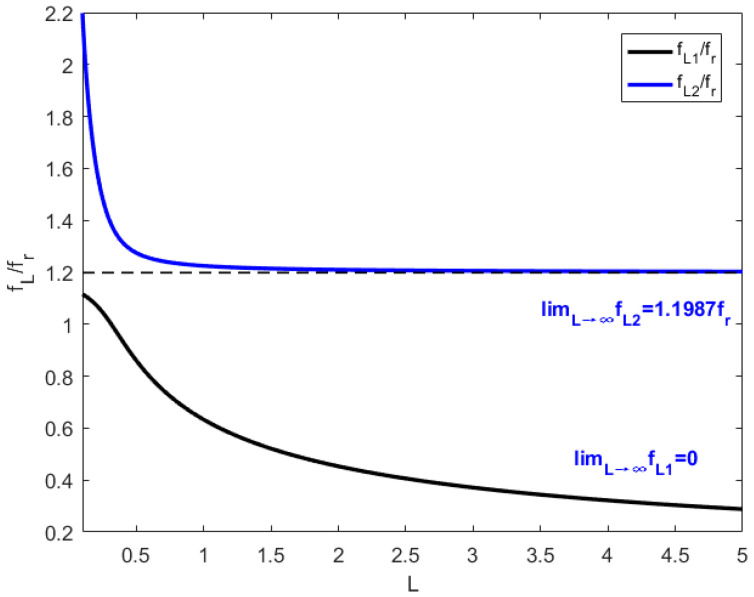
The changes of $f_{L1}$ and $f_{L2}$ with inductance increasing.

Figure 15 shows the change of the piezoelectric resonance frequencies when an inductance is involved in a shunt circuit, which can be derived from Equations (49) and (50). Equations (53) and (54) can be deduced when L approaches ∞.

(53) $$\underset{L\to \infty }{\lim}f_{L1}=0\;\mathrm{Hz}$$

(54) $$\begin{array}{l} \underset{L\to \infty }{\lim}f_{L2}=(-\frac{E_{\mathrm{eq}}H_{\mathrm{eq}}^{3}C_{p}\left(s_{11}^{E}{}^{2}-s_{12}^{E}{}^{2}\right)}{\left(1-\nu_{\mathrm{eq}}^{2}\right)\left(1-\alpha \right)\left({\left(H_{\mathrm{eq}}+2h_{p}\right)}^{3}-{\left(H_{\mathrm{eq}}\right)}^{3}\right)}\\ \;\;\;+\frac{2d_{31}^{2}A_{p}E_{\mathrm{eq}}H_{\mathrm{eq}}^{3}\left(s_{11}^{E}-s_{12}^{E}\right)}{h_{p}\left(1-\nu_{\mathrm{eq}}^{2}\right)\left(1-\alpha \right)\left({\left(H_{\mathrm{eq}}+2h_{p}\right)}^{3}-{\left(H_{\mathrm{eq}}\right)}^{3}\right)}-s_{11}^{E}C_{p}+\frac{d_{31}^{2}A_{p}}{h_{p}})\\ \;\;\;/(\frac{4\pi d_{31}^{2}A_{p}E_{\mathrm{eq}}H_{\mathrm{eq}}^{3}\left(s_{11}^{E}-s_{12}^{E}\right)}{h_{p}\omega_{r}^{2}\left(1-\nu_{\mathrm{eq}}^{2}\right)\left(1-\alpha \right)\left({\left(H_{\mathrm{eq}}+2h_{p}\right)}^{3}-{\left(H_{\mathrm{eq}}\right)}^{3}\right)}\\ \;\;\;-\frac{2\pi C_{p}E_{\mathrm{eq}}H_{\mathrm{eq}}^{3}\left(s_{11}^{E}{}^{2}-s_{12}^{E}{}^{2}\right)}{\omega_{r}^{2}\left(1-\nu_{\mathrm{eq}}^{2}\right)\left(1-\alpha \right)\left({\left(H_{\mathrm{eq}}+2h_{p}\right)}^{3}-{\left(H_{\mathrm{eq}}\right)}^{3}\right)})\mathrm{Hz} \end{array}$$

Substitute the parameter L=0.642 H into Equations (53) and (54), $f_{L2}$ equals $1.1987f_{r}$, and $f_{r}$, equals 1775 Hz. According to Figure 15 as well as Equations (49), (50), (53), (54) and (A4)–(A6), the piezoelectric resonance frequencies $f_{L1}$ and $f_{L2}$ decrease as the increase of the inductance value and eventually stabilize. 

The piezoelectric resonance frequency $f_{L1}$ decreases as the increase of the inductance value. When the inductance approaches being infinitely large, the resonance frequency $f_{L1}$ becomes 0 Hz. However, the other piezoelectric resonance frequency $f_{L2}$ decreases dramatically with the inductance value increase as L is less than 0.4 H. Considering L is larger than 0.4, the drop of piezoelectric resonance frequency $f_{L2}$ slows and $f_{L2}$ stabilizes at $1.1987f_{r}$. The metamaterial plate can generate tunable multiple resonance frequencies when only an inductance is involved in shunting circuit, which has broad application prospects in the field of sound insulation designs in specific frequency bands.

#### 5.2.2. The Capacitance Shunting Circuit

The resonant frequencies when a capacitance is involved in shunting circuit are $f_{r}$ and $f_{C1}$, which equals $\omega_{r}/2\pi$ and $\frac{\omega_{C1}}{2\pi },$ respectively. According to Equation (11), the value of $f_{r}$ can be obtained.

Let $Z=i/\omega_{C1}C$, Equation (A2) can be transformed into Equations (55) and (56), which is the explicit equation of $f_{C1}$.

(55) $$\omega_{C1}^{2}=\omega_{r}^{2}\left(1+\frac{\left(1-\nu_{\mathrm{eq}}^{2}\right)\left(1-\alpha \right)\left({\left(H_{\mathrm{eq}}+2h_{p}\right)}^{3}-{\left(H_{\mathrm{eq}}\right)}^{3}\right)\left(s_{11}^{E}\left(1-\frac{C_{p}}{C}\right)+\frac{d_{31}^{2}A_{p}}{h_{p}C}\right)}{E_{\mathrm{eq}}H_{\mathrm{eq}}^{3}\left(\left(s_{11}^{E}{}^{2}-s_{12}^{E}{}^{2}\right)\left(1-\frac{C_{p}}{C}\right)+\left(s_{11}^{E}-s_{12}^{E}\right)\frac{2d_{31}^{2}A_{p}}{h_{p}C}\right)}\right)$$

(56) $$f_{C1}=f_{r}\sqrt{1+\frac{\left(1-\nu_{\mathrm{eq}}^{2}\right)\left(1-\alpha \right)\left({\left(H_{\mathrm{eq}}+2h_{p}\right)}^{3}-{\left(H_{\mathrm{eq}}\right)}^{3}\right)\left(s_{11}^{E}\left(1-\frac{C_{p}}{C}\right)+\frac{d_{31}^{2}A_{p}}{h_{p}C}\right)}{E_{\mathrm{eq}}H_{\mathrm{eq}}^{3}\left(\left(s_{11}^{E}{}^{2}-s_{12}^{E}{}^{2}\right)\left(1-\frac{C_{p}}{C}\right)+\left(s_{11}^{E}-s_{12}^{E}\right)\frac{2d_{31}^{2}A_{p}}{h_{p}C}\right)}}$$

With the values of the variables in Equations (55) and (56), the value of $f_{C1}$ is obtained, which equals 2103 Hz and confirms the simulation results.

**Figure 16:**
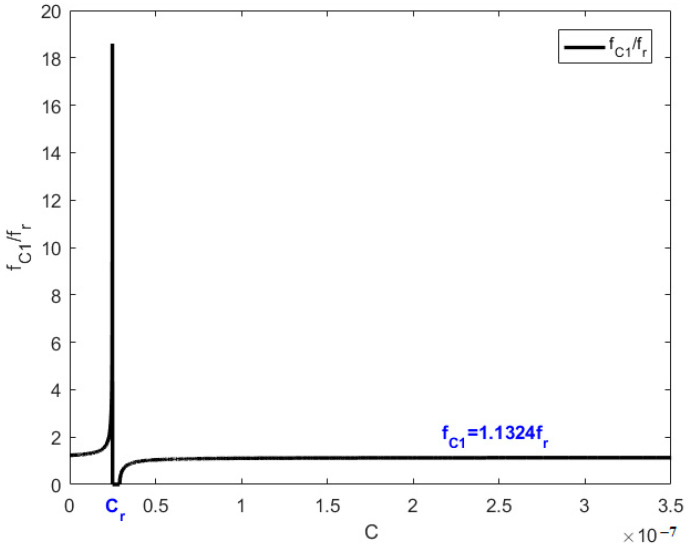
The change of $f_{C1}$ with capacitance increasing.

Figure 16 describes the way in which the piezoelectric resonance frequency $f_{C1}$ with the value of the capacitance C when a capacitance is involved in the shunting circuit. The piezoelectric resonance frequency $f_{C1}$ stabilizes at $1.1324f_{r}$ when capacitance C is not near a fixed value $C_{r}$. When capacitance C is near $C_{r}$, the piezoelectric resonance frequency $f_{C1}$ can vary significantly up to $18f_{r}$. This capacitance value $C_{r}$ can be derived from Equation (56).

(57) $$\left(s_{11}^{E}{}^{2}-s_{12}^{E}{}^{2}\right)\left(1-\frac{C_{p}}{C_{r}}\right)+\left(s_{11}^{E}-s_{12}^{E}\right)\frac{2d_{31}^{2}A_{p}}{h_{p}C_{r}}=0$$

$C_{r}$ can be obtained as

(58) $$C_{r}=C_{p}-\frac{2d_{31}^{2}A_{p}}{h_{p}\left(s_{11}^{E}+s_{12}^{E}\right)}$$

with the actual values of the variables in Equation (58), $C_{r}$ equals $2.4878\times 10^{-8}F$.

#### 5.2.3. The Resistance Shunting Circuit

When resistance is involved in a shunting circle, the resonance frequencies of metamaterial are $f_{r}$ and $f_{R1}$, which equal $\omega_{r}/2\pi$ and $\omega_{R1}/2\pi$, respectively. The mass resonance frequency $f_{r}$ can be obtained by Equation (11).

With $Z_{i}=iR$, Equation (A2) can be converted to Equations (59) and (60), which is the explicit equation of $f_{R1}$.

(59) $$\omega_{R1}^{2}=\omega_{r}^{2}\left(1+\frac{s_{11}^{E}\left(1-\nu_{\mathrm{eq}}^{2}\right)\left(1-\alpha \right)\left({\left(H_{\mathrm{eq}}+2h_{p}\right)}^{3}-{\left(H_{\mathrm{eq}}\right)}^{3}\right)}{\left(s_{11}^{E}{}^{2}-s_{12}^{E}{}^{2}\right)E_{\mathrm{eq}}H_{\mathrm{eq}}^{3}}\right)$$

(60) $$f_{R1}=f_{r}\sqrt{1+\frac{s_{11}^{E}\left(1-\nu_{\mathrm{eq}}^{2}\right)\left(1-\alpha \right)\left({\left(H_{\mathrm{eq}}+2h_{p}\right)}^{3}-{\left(H_{\mathrm{eq}}\right)}^{3}\right)}{\left(s_{11}^{E}{}^{2}-s_{12}^{E}{}^{2}\right)E_{\mathrm{eq}}H_{\mathrm{eq}}^{3}}}$$

**Figure 17:**
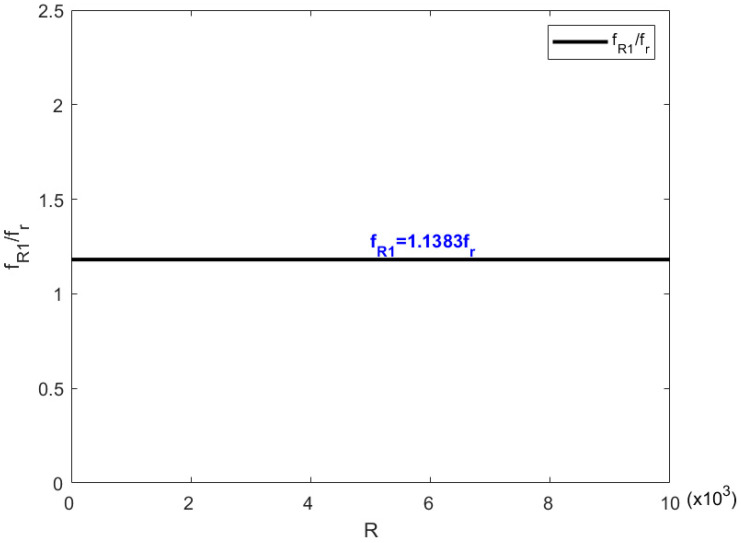
The change in $f_{R1}$ with the resistance increasing.

Figure 17 and Equation (60) both prove that the piezoelectric resonance frequency $f_{R1}$ is a constant and is independent of the resistance value when a resistance is involved in a shunting circle. With the actual values of the variables in Equation (60), the value of $f_{R1}$ can be obtained and equals $1.1383f_{r}=1685\;\mathrm{Hz}$, which confirms the simulation results.

### 5.3. Effect of the Area Ratio of the Piezoelectric Patches

**Figure 18:**
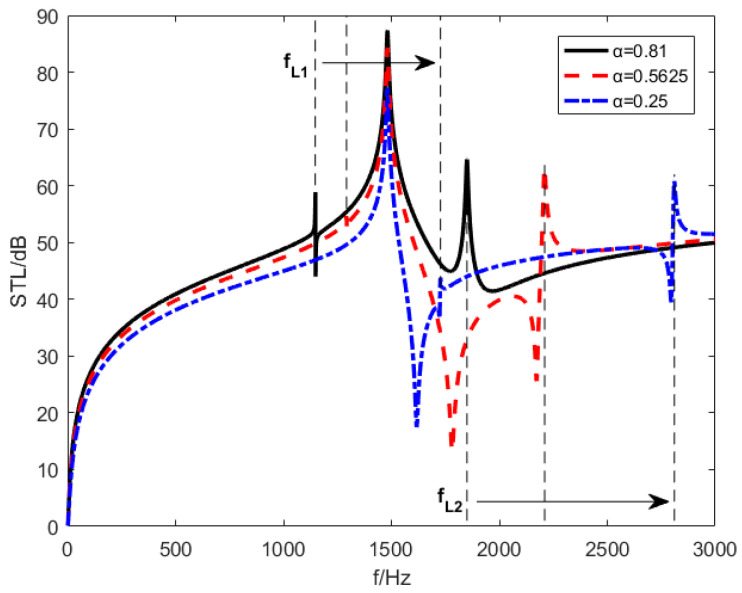
STL curves under different piezoelectric sheet area ratios.

With the same elevation angle $\theta =30^{\circ }$ and the same inductance shunting circuit, Figure 18 shows the STL curves under different piezoelectric-sheet-area–substrate-area ratios α. The piezoelectric-patch-area–substrate-area ratio α has three distinct values: 0.81, 0.5625 and 0.25. The results show that the frequencies $f_{L1}$ and $f_{L2}$ gradually become larger with decreasing of the area ratio of piezoelectric sheets. $f_{L1}$ and $f_{L2}$ changing from the same side to the opposite side of $f_{r}$. Simultaneously, with the reduction of the piezoelectric sheet area ratio, the resonance frequencies bandwidth of mass resonance frequencies $f_{r}$ becomes narrower. The STL amplitudes of the piezoelectric resonance frequencies $f_{L1}$ and $f_{L2}$ are reduced at the same time.

**Figure 19:**
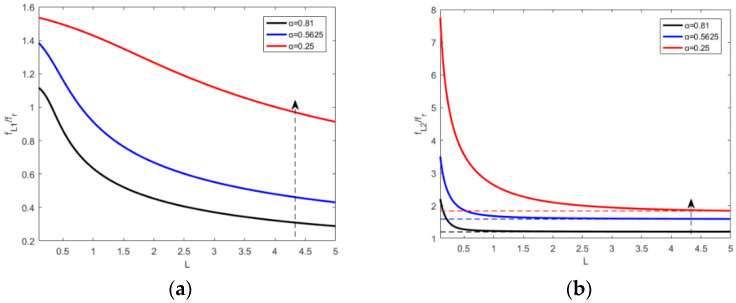
The change of piezoelectric resonance frequencies with inductance value. (**a**) $f_{L1}$ and (**b**) $f_{L2}$.

Figure 19 indicates the effect of area ratio on the control law of inductance when an inductance is involved in shunting circuit. At the same time, it can be seen from Equation (49) that the area of the piezoelectric sheet has no effect on the limit value of $f_{L1}$, which is still 0 Hz. However, area ratio determines the lowest tuning limit of $f_{L2}$, which means that the larger area ratio of the piezoelectric sheet, piezoelectric resonance frequency $f_{L2}$ can be tuned to a lower frequency by increasing the inductance value.

Considering the influence characteristics of the above key factors, the high value of STL in broadband can be achieved by setting the incidence angle reasonably. High STL values can be transferred to low frequencies by increasing the inductance value or area ratio.

## 6. Conclusions

In this paper, the STL of a metamaterial honeycomb core sandwich plate was studied. The designed plate had piezoelectric sheets arranged in sub-wavelength cycles and elastically connected on each side. In order to predict the STL, the effective medium (EM) method was utilized to treat the metamaterial plate as a homogeneous plate with effective dynamic mass density and effective dynamic bending stiffness. 

A theoretical model for the numerical calculation of oblique and diffuse-field STL was established using the structural bending wave method, which was compared with the simulation of the acoustic-structure–piezoelectric-coupling model. The numerical results show that metamaterial plates could produce three significant resonance frequencies in an oblique STL curve or diffuse-field curve, which were significantly better than a metamaterial with rigidly connected piezoelectric sheet or bare plates with the same mass in some bands, and the piezoelectric resonance frequencies were closely related to the inductance values, shunting circuit and piezoelectric sheet area ratios.

When sound is incident vertically, there is only one mass resonance frequency, and anti-resonance mode is exhibited. Considering that sound is incident obliquely or in diffuse fields, two new piezoelectric resonance frequencies are generated, while anti-resonance is suppressed. As the elevation angle increases, the piezoelectric resonance frequencies bands become wider but the mass resonance frequency band becomes narrower. The sound insulation effect in other frequency bands is sacrificed.

As an inductance is involved in a shunting circuit, a metamaterial plate exhibits three resonance frequencies, and the piezoelectric resonance frequencies decrease with the increase of the inductance value and tend to be stable. When a capacitance or resistance is involved in a shunting circuit, the metamaterial plate exhibits two resonance frequencies that are not easy to tune but are valuable for complex shunt circuit designing. Piezoelectric resonance frequencies gradually increase as the area ratio decreases, and they transition from being on the opposite side of $f_{r}$ to being on the same side of $f_{r}$. The bandwidth of the mass resonance frequency $f_{r}$ is narrowed.

## Figures and Tables

**Table 1:** Honeycomb sandwich panel parameters.

$a_{0}\left(mm\right)$	$b_{0}\left(mm\right)$	$h_{c}\left(mm\right)$	$h_{f}\left(mm\right)$	$E_{f}\left(GPa\right)$
** 6 **	1	2	1	200
** $\rho_{f}\left(kg/m^{3}\right)$ **	$\rho_{c}\left(\mathrm{kg}/m^{3}\right)$	$\nu_{f}$	$a_{x}$	$a_{y}$
** 8960 **	8960	0.32	$4\sqrt{3}a_{0}+4b_{0}$	$6a_{0}+2\sqrt{3}b_{0}$

**Table 2:** Piezoelectric sheet parameters.

$l_{px}\left(mm\right)$	$l_{py}\left(mm\right)$	$h_{p}\left(mm\right)$	$\rho_{p}\left(kg/m^{3}\right)$
** 41 **	35	1	7500
$s_{11}^{E}\left(m^{2}/N\right)$	$s_{12}^{E}\left(m^{2}/N\right)$	$d_{31}\left(C/N\right)$	$\varepsilon_{33}^{T}\left(F/m\right)$
** $16.5\times 10^{-12}$ **	$-4.78\times 10^{-12}$	$-2.74\times 10^{-10}$	$3.01\times 10^{-8}$

## Data Availability

The data used to support the findings of this study are included within the article.

## Appendix A

Explicit functions of $f_{L1}$ and $f_{L2}$ are derived and Equation (A1) can be obtained from the condition that the effective dynamic bending stiffness $D_{\mathrm{eff}}$ approaches 0, which is introduced in Equation (33).

(A1) $$\left(1-\alpha \right)D_{A}+\alpha D_{B}=0$$

Equation (A2) can be obtained when Equation (A1) is introduced in Equations (30) and (31).

(A2) $$\frac{E_{\mathrm{eq}}H_{\mathrm{eq}}^{3}}{12\left(1-\nu_{\mathrm{eq}}^{2}\right)}+\left(1-\alpha \right)\frac{2E_{p}}{3\left(1-\nu_{p}^{2}\right)}\left[{\left(\frac{H_{\mathrm{eq}}}{2}+h_{p}\right)}^{3}-{\left(\frac{H_{\mathrm{eq}}}{2}\right)}^{3}\right]\left[\frac{1}{1-\omega^{2}/\left[\omega_{r}^{2}\left(1+i\eta_{r}\right)\right]}\right]=0$$

Equation (A2) is introduced in Equations (26) and (27), and s=iω and Z=iωL.

(A3) $$\begin{array}{l} \omega^{4}(\frac{2Ld_{31}^{2}A_{p}E_{\mathrm{eq}}H_{\mathrm{eq}}^{3}\left(s_{11}^{E}-s_{12}^{E}\right)}{h_{p}\omega_{r}^{2}\left(1-\nu_{\mathrm{eq}}^{2}\right)\left(1-\alpha \right)\left({\left(H_{\mathrm{eq}}+2h_{p}\right)}^{3}-{\left(H_{\mathrm{eq}}\right)}^{3}\right)}\\ \;\;-\frac{LC_{p}E_{\mathrm{eq}}H_{\mathrm{eq}}^{3}\left(s_{11}^{E}{}^{2}-s_{12}^{E}{}^{2}\right)}{\omega_{r}^{2}\left(1-\nu_{\mathrm{eq}}^{2}\right)\left(1-\alpha \right)\left({\left(H_{\mathrm{eq}}+2h_{p}\right)}^{3}-{\left(H_{\mathrm{eq}}\right)}^{3}\right)})\\ \;\;+\omega^{2}(\frac{E_{\mathrm{eq}}H_{\mathrm{eq}}^{3}\left(s_{11}^{E}{}^{2}-s_{12}^{E}{}^{2}\right)}{\omega_{r}^{2}\left(1-\nu_{\mathrm{eq}}^{2}\right)\left(1-\alpha \right)\left({\left(H_{\mathrm{eq}}+2h_{p}\right)}^{3}-{\left(H_{\mathrm{eq}}\right)}^{3}\right)}\\ \;\;+\frac{E_{\mathrm{eq}}H_{\mathrm{eq}}^{3}LC_{p}\left(s_{11}^{E}{}^{2}-s_{12}^{E}{}^{2}\right)}{\left(1-\nu_{\mathrm{eq}}^{2}\right)\left(1-\alpha \right)\left({\left(H_{\mathrm{eq}}+2h_{p}\right)}^{3}-{\left(H_{\mathrm{eq}}\right)}^{3}\right)}\\ \;\;-\frac{2Ld_{31}^{2}A_{p}E_{\mathrm{eq}}H_{\mathrm{eq}}^{3}\left(s_{11}^{E}-s_{12}^{E}\right)}{h_{p}\left(1-\nu_{\mathrm{eq}}^{2}\right)\left(1-\alpha \right)\left({\left(H_{\mathrm{eq}}+2h_{p}\right)}^{3}-{\left(H_{\mathrm{eq}}\right)}^{3}\right)}+s_{11}^{E}LC_{p}\\ \;\;-\frac{Ld_{31}^{2}A_{p}}{h_{p}})\\ \;\;-\left(s_{11}^{E}+\frac{E_{\mathrm{eq}}H_{\mathrm{eq}}^{3}\left(s_{11}^{E}{}^{2}-s_{12}^{E}{}^{2}\right)}{\left(1-\nu_{\mathrm{eq}}^{2}\right)\left(1-\alpha \right)\left({\left(H_{\mathrm{eq}}+2h_{p}\right)}^{3}-{\left(H_{\mathrm{eq}}\right)}^{3}\right)}\right)=0 \end{array}$$

Equation (A3) is a univariate quadratic equation where $\omega^{2}$ is the variable, and it can be simplified to $X_{1}\omega^{4}+X_{2}\omega^{2}+X_{3}=0$. Equations (A4)–(A6) describe $X_{1}$, $X_{2}$ and $X_{3}$, respectively.

(A4) $$\begin{array}{c} X_{1}=\frac{2Ld_{31}^{2}A_{p}E_{\mathrm{eq}}H_{\mathrm{eq}}^{3}\left(s_{11}^{E}-s_{12}^{E}\right)}{h_{p}\omega_{r}^{2}\left(1-\nu_{\mathrm{eq}}^{2}\right)\left(1-\alpha \right)\left({\left(H_{\mathrm{eq}}+2h_{p}\right)}^{3}-{\left(H_{\mathrm{eq}}\right)}^{3}\right)}\\ \;-\frac{LC_{p}E_{\mathrm{eq}}H_{\mathrm{eq}}^{3}\left(s_{11}^{E}{}^{2}-s_{12}^{E}{}^{2}\right)}{\omega_{r}^{2}\left(1-\nu_{\mathrm{eq}}^{2}\right)\left(1-\alpha \right)\left({\left(H_{\mathrm{eq}}+2h_{p}\right)}^{3}-{\left(H_{\mathrm{eq}}\right)}^{3}\right)} \end{array}$$

(A5) $$\begin{array}{ll} X_{2}= & \frac{E_{\mathrm{eq}}H_{\mathrm{eq}}^{3}\left(s_{11}^{E}{}^{2}-s_{12}^{E}{}^{2}\right)}{\omega_{r}^{2}\left(1-\nu_{\mathrm{eq}}^{2}\right)\left(1-\alpha \right)\left({\left(H_{\mathrm{eq}}+2h_{p}\right)}^{3}-{\left(H_{\mathrm{eq}}\right)}^{3}\right)}\\ & \;+\frac{E_{\mathrm{eq}}H_{\mathrm{eq}}^{3}LC_{p}\left(s_{11}^{E}{}^{2}-s_{12}^{E}{}^{2}\right)}{\left(1-\nu_{\mathrm{eq}}^{2}\right)\left(1-\alpha \right)\left({\left(H_{\mathrm{eq}}+2h_{p}\right)}^{3}-{\left(H_{\mathrm{eq}}\right)}^{3}\right)}\\ & \;-\frac{2Ld_{31}^{2}A_{p}E_{\mathrm{eq}}H_{\mathrm{eq}}^{3}\left(s_{11}^{E}-s_{12}^{E}\right)}{h_{p}\left(1-\nu_{\mathrm{eq}}^{2}\right)\left(1-\alpha \right)\left({\left(H_{\mathrm{eq}}+2h_{p}\right)}^{3}-{\left(H_{\mathrm{eq}}\right)}^{3}\right)}+s_{11}^{E}LC_{p}\;\\ & \;-\frac{Ld_{31}^{2}A_{p}}{h_{p}} \end{array}$$

(A6) $$X_{3}=-\left(s_{11}^{E}+\frac{E_{\mathrm{eq}}H_{\mathrm{eq}}^{3}\left(s_{11}^{E}{}^{2}-s_{12}^{E}{}^{2}\right)}{\left(1-\nu_{\mathrm{eq}}^{2}\right)\left(1-\alpha \right)\left({\left(H_{\mathrm{eq}}+2h_{p}\right)}^{3}-{\left(H_{\mathrm{eq}}\right)}^{3}\right)}\right)$$
